# Functional plasticity of the swim bladder as an acoustic organ for communication in a vocal fish

**DOI:** 10.1098/rspb.2023.1839

**Published:** 2023-12-13

**Authors:** Loranzie S. Rogers, Nicholas R. Lozier, Yulia P. Sapozhnikova, Kelly M. Diamond, Julian Ly Davis, Joseph A. Sisneros

**Affiliations:** ^1^ Department of Psychology, University of Washington, Seattle, WA, USA; ^2^ Laboratory of Ichthyology, Limnological Institute Siberian Branch of the Russian Academy of Sciences, Irkutsk, Russia; ^3^ Center for Developmental Biology and Regenerative Medicine, Seattle Children's Research Institute, Seattle, WA, USA; ^4^ Department of Engineering, University of Southern Indiana, Evansville, IN, USA; ^5^ Virginia Merrill Bloedel Hearing Research Center, University of Washington, Seattle, WA, USA; ^6^ Department of Biology, University of Washington, Seattle, WA, USA

**Keywords:** plasticity, swim bladder, fish hearing, sound production, sound pressure

## Abstract

Teleost fishes have evolved a number of sound-producing mechanisms, including vibrations of the swim bladder. In addition to sound production, the swim bladder also aids in sound reception. While the production and reception of sound by the swim bladder has been described separately in fishes, the extent to which it operates for both in a single species is unknown. Here, using morphological, electrophysiological and modelling approaches, we show that the swim bladder of male plainfin midshipman fish (*Porichthys notatus*) exhibits reproductive state-dependent changes in morphology and function for sound production and reception. Non-reproductive males possess rostral ‘horn-like’ swim bladder extensions that enhance low-frequency (less than 800 Hz) sound pressure sensitivity by decreasing the distance between the swim bladder and inner ear, thus enabling pressure-induced swim bladder vibrations to be transduced to the inner ear. By contrast, reproductive males display enlarged swim bladder sonic muscles that enable the production of advertisement calls but also alter swim bladder morphology and increase the swim bladder to inner ear distance, effectively reducing sound pressure sensitivity. Taken together, we show that the swim bladder exhibits a seasonal functional plasticity that allows it to effectively mediate both the production and reception of sound in a vocal teleost fish.

## 1. Introduction

Vocal-acoustic communication necessitates the production and reception of acoustic signals. The maintenance of such communication systems requires that sender and receiver systems co-evolve within the constraints of their ecological niche [1]. Among many terrestrial and semi-aquatic vertebrates, the organs for vocal production (i.e. larynx or syrinx) and auditory reception (e.g. cochlea or basilar papillae) are separate and distinct. Yet these sender and receiver systems often mutually evolve through natural selection to enable the transmission of biologically relevant information that benefits both sender and receiver. Perhaps the most notable biologically relevant signals produced by animals are the acoustic advertisement calls generated by males to attract females for reproduction (e.g. rodents [2,3], birds [4,5] and frogs [6,7]). These social acoustic signals are detected by the ear in the form of sound pressure signals, which are then transduced by the tympanic middle ear in mammals, birds, and anurans to fluid-membrane motion within the inner ear resulting in hair cell stimulation and further activation of the auditory system [8].

By contrast to terrestrial and semi-aquatic vertebrates, the ear of teleost fishes, sharks, skates and rays is designed to detect acoustic particle motion (i.e. particle displacement, velocity or acceleration). All fishes are posited to detect particle motion via their otolithic inner ear end organs (saccule, utricle and lagena), which respond to direct displacement and linear acceleration. However, a subset of fishes (e.g. Ostariophysan and Gadiform fishes) have evolved the ability to detect sound pressure [9–11]. For this subset of more recently derived fishes, a number of sound pressure detection mechanisms that effectively enhance fish auditory sensitivity have been described (e.g. [12–15]). The most common mechanism involves the swim bladder, which in addition to regulating buoyancy, serving as an oxygen reservoir, and facilitating sound production, also aids in the reception of sound pressure signals (for review, see [16–18]). Whereby, impinging sound pressure waves induce vibrations of the receiver's swim bladder, which generates local particle motion that the inner ear otolithic end organs can detect. The degree of pressure sensitivity for a given fish species is directly related to the proximity of the swim bladder to the auditory inner ear [9–11]. However, previous work has shown that the position of the swim bladder relative to the inner ear is sex dependent in a number of vocal fishes that display sexually dimorphic differences in swim bladder morphology. These sex differences in swim bladder morphology are indirectly related to sexual dimorphisms in sonic muscle morphology and are posited to result in sexually dimorphic differences in sound pressure sensitivity [19]. Interestingly, among many vocal fishes, sonic muscles undergo seasonal hypertrophy [20–23], which may, in turn, influence the swim bladder-inner ear distance relationship and affect sound pressure sensitivity.

**Figure 1.**
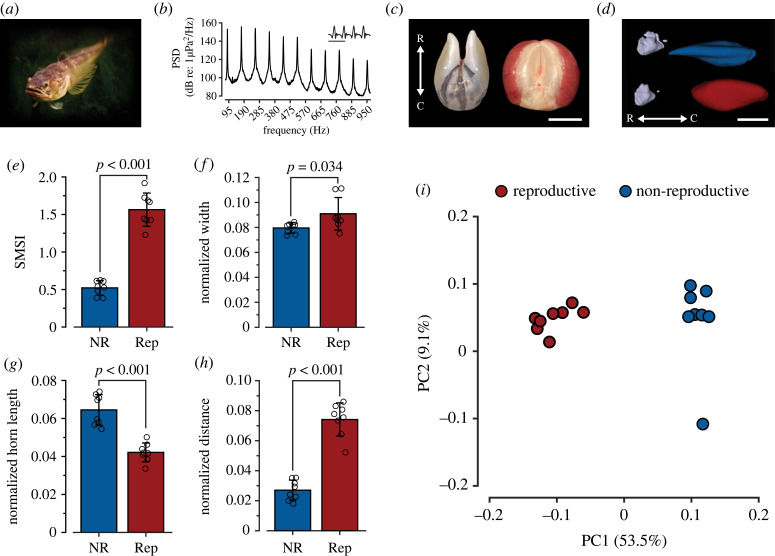
Male plainfin midshipman sonic muscles exhibit seasonal changes that result in seasonally dimorphic swim bladder morphology. (*a*) Plainfin midshipman (*Porichthys notatus*). (*b*) Power spectral density (PSD) curve (dB re: 1 µPa^2^/Hz) of type I male midshipman advertisement call. *Inset:* waveform of hum. Scale bar represents 10 ms. (*c*) Dorsal view of dissected non-reproductive (left) and reproductive (right) type I male midshipman swim bladders. Scale bar represents 1 cm. (*d*) Representative images that show the diversity in non-reproductive (blue) and reproductive (red) swim bladder morphology and relationship to the inner ear. Scale bar represents 1 cm. (*e*) Sonic muscle somatic index of non-reproductive (NR, blue) and reproductive (Rep, red) type I male midshipman. *n* = 8/group, *p* < 0.001, two-tailed Student's *t*-test. (*f*) Normalized width of non-reproductive (NR, blue) and reproductive (Rep, red) type I male midshipman swim bladders. *n* = 8/group, *p* = 0.035, two-tailed Student's *t*-test. (*g*) Normalized horn length of non-reproductive (NR, blue) and reproductive (Rep, red) type I male midshipman swim bladders. *n* = 8/group, *p* < 0.001, two-tailed Student's *t*-test. (*h*) Normalized distance between swim bladder horns and the saccule of non-reproductive (NR, blue) and reproductive (Rep, red) type I male midshipman. *n* = 8/group, *p* < 0.001, two-tailed Student's *t*-test. Measurements were normalized for the size (length) by dividing each specific measurement by the animal's standard length. These normalized morphometric measurements were then used to examine reproductive state differences in male swim bladder morphology. All error bars represent ± 1 s.d. (*i*) Morphospace from pseudo-landmark principle component analysis (PCA) of non-reproductive (blue) and reproductive (red) type I male midshipman swim bladders.

Some of the most extensively studied fishes with regard to acoustic communication are found in the family Batrachoididae (toadfishes and midshipman fish) [24–29]. Plainfin midshipman (*Porichthys notatus*; figure 1*a*) are a seasonally breeding fish that are highly dependent upon the production and reception of acoustic signals for intraspecific communication during the reproductive season. Thus, the reliance upon sound for communication during social behaviours makes midshipman a tractable model for investigating the neural mechanisms of acoustic communication, especially those related to seasonal changes in the production and reception of social acoustic signals [30–35]. During late spring and summer, courting (type I) males establish nest sites in the rocky intertidal substrate and produce long-duration, multiharmonic advertisement calls (figure 1*b*) to attract gravid females for reproduction [36]. Prior to the summer breeding season, type I males undergo seasonal, androgen-dependent hypertrophy of the swim bladder sonic muscles, which are used to produce the seasonal advertisement (i.e. mate) calls [21]. As a result, the sonic muscles of reproductive type I males become approximately 7× and 10× larger than that of reproductive females and type II males, respectively [21,28,37,38]. Subsequently, reproductive midshipman display sexually dimorphic differences in swim bladder morphology, where females and type II males exhibit elongated rostral extensions that bring the swim bladder closer in proximity to the inner ear than in type I males [19]. Furthermore, these rostral swim bladder extensions function to enhance the sound pressure and frequency sensitivity of auditory hair cells in the saccule and lagena of females [39,40]. Conversely, type I males are posited to lack or have reduced sound pressure sensitivity due to the absence of rostral swim bladder extensions, which is attributed to their enlarged sonic muscles used to produce advertisement calls [19,39]. However, during the non-breeding season, type I male swim bladder sonic muscles are known to atrophy [21]. Whether such seasonal changes in type I male sonic muscle morphology affects the swim bladder-inner ear distance relationship and sensitivity to sound pressure remains to be determined.

Here, we investigate whether sound pressure sensitivity in type I male midshipman is seasonally modulated by morphometric changes of the swim bladder due to seasonal hypertrophy and atrophy of the sonic muscles. We test the hypothesis that seasonal variation in type I male swim bladder morphology, caused by changes in the sonic muscles, results in seasonal changes in the functionality of the swim bladder as an acoustic organ for communication. We predict that reproductive type I male swim bladder morphology is well suited for advertisement call (sound) production, while in non-reproductive type I males, the swim bladder morphology aids in detecting sound pressure signals (hearing). We show, using microCT scanning, auditory electrophysiology and finite-element modelling, that type I male plainfin midshipman exhibit novel, seasonal changes in the morphology and function of the swim bladder to effectively enhance acoustic communication. Our study demonstrates that the swim bladder in type I male midshipman can operate in the reproductive season as a sound production organ to generate acoustic advertisement calls, and in the non-reproductive season as an accessory hearing organ to enhance sound pressure sensitivity by increasing the gain and frequency sensitivity of auditory saccular hair cells. Thus, we show that the functionality of the swim bladder changes seasonally to aid in mediating both sound production and hearing in a vocal fish species.

## 2. Material and methods

### (a) Animals

Adult male midshipman were collected from the Puget Sound during the non-reproductive (February 2022) and reproductive breeding season (May–June 2022). Fish were transferred to the University of Washington, housed in recirculating artificial saltwater tanks, and subjected to either non-reproductive (9/15 h) or reproductive (12/12 h) light/dark photoperiods. All experiments were performed within 21 and 14 days of collection during the non-reproductive and reproductive seasons, respectively, which allowed for animals to recover from capture-related stress while minimizing any effects prolonged captivity may have on auditory sensitivity.

Standard length (cm), body mass (g), sex and reproductive state, which was determined by evaluating the gonadosomatic index (100 * (gonad mass/(body mass – gonad mass))), were recorded for all fish. All animal care and experimental procedures were approved by the University of Washington Institutional Animal Care and Use Committee (Protocol ID: 4079-01) and conformed to the NIH *Guide for the Care and Use of Laboratory Animals*.

### (b) Physiology

**Figure 2.**
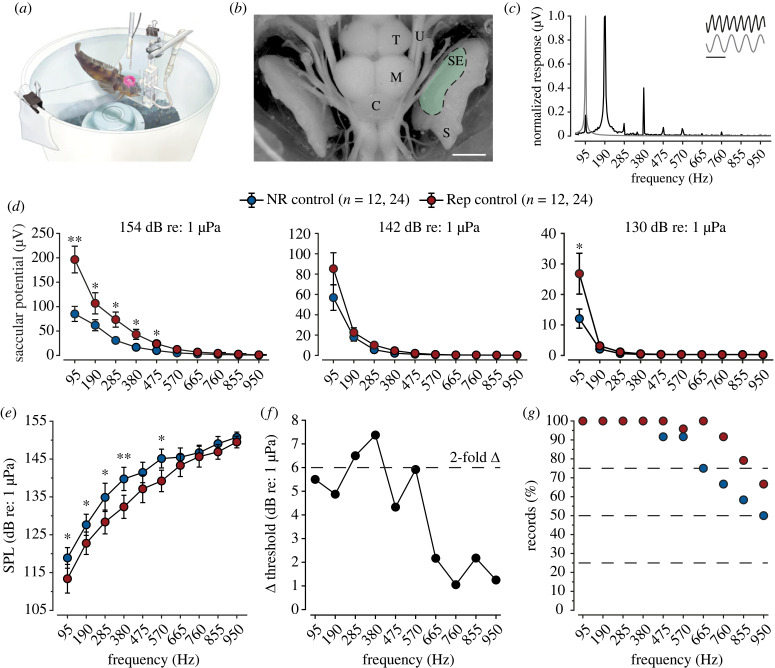
Male midshipman saccular hair cell auditory sensitivity is seasonally dependent. (*a*) Illustration of electrophysiology test tank. (*b*) Dorsal view of midshipman brain and inner ear. The shaded green region represents the saccular sensory epithelium (SE), and the location of hair cell evoked potential recordings. Abbreviations: *S,* saccule; *U,* utricle; *C,* cerebellum; *M,* midbrain and *T,* telencephalon. The scale bar represents 5 mm. (*c*) Normalized saccular hair cell evoked response (black) to a 95 Hz tone (grey). Scale represents 10 ms. (*d*) Non-reproductive (blue) and reproductive (red) male plainfin midshipman saccular hair cell iso-level response profiles at three representative sound pressure levels (dB re: 1 µPa). Data are plotted as mean ± 1 SE. (*e*) Non-reproductive (blue) and reproductive (red) male midshipman saccular hair cell auditory threshold tuning curves relative to sound pressure level (dB re: 1 µPa). All data are plotted as mean ± 95% confidence interval. Asterisks indicate significant differences across frequencies: *p < 0.05, **p < 0.01. The number of animals and records for each group is indicated in parentheses. (*f*) Saccular hair cell sound pressure threshold difference (Δ dB re: 1 µPa) between reproductive and non-reproductive male midshipman. The dashed line indicates a twofold change in auditory sensitivity. (*g*) Percentage of non-reproductive (blue) and reproductive (red) male midshipman evoked potential recordings that displayed thresholds above background noise levels across the bandwidth of test frequencies.

The methodology for conducting *in vivo* auditory evoked hair cell potentials in midshipman closely follows the techniques used in prior studies [33,39,41–44]. Briefly, a small incision, approximately 1.5 cm in length, was made on the ventral surface, approximately 1 cm rostral to the vent, of anaesthetized fish near the location of the swim bladder to remove the connective tissue and swim bladder (figure 1*c*) from the body cavity. Following removal, the incision was sealed with continuous sutures. Alternatively, control fish underwent a sham surgery, which followed the same surgical protocol except for the removal of the swim bladder. Following surgical manipulations, a bilateral craniotomy was performed to expose both left and right inner ear saccules, and a hydrophobic barrier (roughly 3 cm diameter × 5 cm height) around the craniotomy was fashioned to create a water-tight seal (figure 2*a*). Fish were then head fixed and suspended 4 cm below the water's surface in the centre of an experimental tank (40 cm diameter, 20 cm water depth) (figure 2*a*) that was situated inside a sound attenuation chamber on a vibration-isolation table. Fish were perfused throughout experimental testing with chilled saltwater (13–15°C).

Auditory evoked saccular hair cell potentials were recorded for populations of saccular hair cells using 3 M KCl glass microelectrodes (impedance: 4.0–8.0 MΩ) positioned near (≤ 2 mm) the saccular sensory epithelia (figure 2*b*, *SE*). Evoked potentials (amplification: 100×, bandpass filter: 0.07 to 3 kHz) were recorded in response to calibrated pure tone signals that were generated using a lock-in amplifier (SR830, Stanford Research Systems) connected to an audio amplifier (BG-1120, TOA Corporation) and then to an underwater speaker (UW-30, Telex Communications) (figure 2*a*). The DC output signal (RMS) recorded in response to auditory stimuli is proportional to the saccular hair cell evoked response to the second harmonic of the stimulus frequency as this corresponds to the maximal evoked potential amplitude due to populations of oppositely oriented teleost inner ear hair cells [41,45,46] (figure 2*c*). Prior to each physiology recording session, background saccular potential levels were measured (*n* = 8) under ambient sound levels in the absence of auditory stimuli (electronic supplementary material, figure S1, *dashed line*). After determining background levels, iso-intensity level profiles were measured in response to randomly presented tones (500 ms duration, 0.25 Hz duty cycle, 8 repetitions, test frequencies: 95, 190, 285, 380, 475, 570, 665, 760, 855 and 950 Hz) that spanned frequencies encompassed within male midshipman advertisement call while avoiding experimental tank resonance frequencies (electronic supplementary material, figure S2). All experimental trials were controlled using custom MATLAB scripts. Saccular hair cell sound pressure level (dB re: 1 µPa) tuning curves were determined in response to the test frequencies (95–950 Hz) over the range of sound levels (100–154 dB re: 1 µPa, 3 dB increments) using a threshold determination technique that required a mean saccular evoked potential at least two standard deviations above background levels (electronic supplementary material, figure S1).

### (c) MicroCT scanning

Midshipman were scanned on a SkyScan 1076 High Energy microCT (Bruker, Inc., USA) with a scan resolution of 17.6 µm/pixel. Prior to scanning, midshipman were sacrificed in a benzocaine bath and then placed in a silicone cylinder stuffed with Kimwipes to fill in negative space and prevent movement during scanning. Care was taken to avoid deformation of the swim bladder, which remained gas-filled for all scans. Digital scan volumes were reconstructed using NRecon software (Micro Photonics Inc., Allentown, PA, USA), and all additional processing and visualizations were performed using three-dimensional Slicer ([47], https://www.slicer.org/).

### (d) Morphometric analyses

All reconstructed microCT images were rendered as three-dimensional volumes using 3D Slicer ([47], https://www.slicer.org/), and individual swim bladder and otolith volumes were segmented using histogram thresholding. Following segmentation, swim bladder width, horn length, which was defined as the difference between the swim bladder length and width, and swim bladder to otolith distance, which was measured bilaterally and averaged within each subject to account for any differences in laterality, were determined (electronic supplementary material, figure S3*a* and figure S3*b*). To account for differences in animal size, all measurements were normalized by dividing by the fish's standard length, thus creating a normalized ratio for all measurements.

To identify additional morphological differences between non-reproductive and reproductive male swim bladders, pseudo-landmark points were generated for one male swim bladder model (i.e. template swim bladder) using SlicerMorph's PseudoLMGenerator module, which generates a dense symmetric set of surface points [48]. Pseudo-landmark points were then validated to ensure all points were placed on the surface of the template swim bladder. The final number of pseudo-landmarks on the template swim bladder was 2483 (electronic supplementary material, figure S3*c*). Pseudo-landmark points for the template swim bladder were then transferred to all other swim bladder models using SlicerMorph's ALPACA module [49]. Following the transfer of pseudo-landmarks to all swim bladder models, principal component analysis (PCA) using SlicerMorph was conducted to determine if there were differences in the swim bladders of males from different reproductive states.

### (e) Finite-element analysis

Models of non-reproductive and reproductive male swim bladders from three-dimensional Slicer were imported as stereolithography files into FreeCad (V0.20.1, https://www.freecad.org). Swim bladders were reoriented to the origin, and dorsal-ventral planes were inserted every 22.5 degrees around the horizontal plane such that swim bladders were split into 16 discrete sections (electronic supplementary material, figure S4*a*). Sectioned swim bladders were then exported to Strand7 (R3.1.1, Strand7 Pty Ltd). Models were then meshed with triangular elements and given material properties (1 MPa) and thickness (824.5 µm) collected from oyster toadfish (*Opsanus tau*), a close relative of the plainfin midshipman [50]. Density was approximated to be close to water (1000 kg m^−3^). For dynamic analysis, swim bladders were considered to be underdamped with a damping ratio of 0.325 [51]. Additionally, boundary conditions were applied to model the attachment of the swim bladder to the internal dorsal surface. Nodes along the dorsal-caudal end of the midline of the swim bladder were fixed (i.e. no displacement and no rotation degrees of freedom).

Next, natural frequencies (*ω*_n_'s), which are the frequencies at which a system oscillates after excitation with no force applied, and displacement mode shapes (*λ*'s), which show the maximal relative displacement of the structure from equilibrium at each natural frequency, were determined from Strand7. Finally, since midshipman often rely on detecting and producing sound in shallow water environments, we modelled pressure impinging the swim bladder in the horizontal plane along the dorsal-ventral midline to determine the relative displacement and power. Measurements were determined in response to a total force of 3.03 × 10^−6^ Newtons over approximately 3.03 × 10^−6^ m^2^ area of elements, such that a global pressure load of 1 Pascal was applied at loading angles ranging from 0° to 315° in 45° increments (electronic supplementary material, figure S4*a*). Displacement and power were determined at all natural frequencies up to 750 Hz, and frequencies from 0–750 Hz in 25 Hz increments. Due to a large number of points, displacement and power were determined at representative points, which were positioned at the rostral horns, and along the dorsal, midline and ventral surface of the swim bladder at each of the 16 sections (electronic supplementary material, figure S4*b*).

### (f) Statistical analysis

All statistical analyses were performed using R software (R Core Team, Vienna, Austria) with the following packages: car, ggpubr, lme4, rstatix and tidyverse. For all statistical tests, a significance level of 0.05 was defined. Differences in swim bladder morphology of different reproductive state males were assessed using two-sample *t*-tests, which were performed to determine significant differences in sonic muscle somatic index (SMSI), swim bladder width and horn length, and swim bladder to otolith distances.

To determine if the swim bladder modulates saccular hair cell auditory thresholds, the effects of swim bladder condition (i.e. control or removal) were analysed via a one-way repeated-measures ANOVA (between-subject factor: swim bladder condition, within-subject factor: stimulus frequency * swim bladder condition). Given that we were only interested in how swim bladder condition modulates frequency sensitivity, *a priori* pairwise *t*-tests compared the frequency-specific auditory sensitivity of non-reproductive and reproductive males across the stimulus frequency bandwidth (95–950 Hz).

## 3. Results

Type I male midshipman exhibited seasonal changes in swim bladder shape and sonic muscle mass. Reproductive males had a sonic muscle-somatic index that was threefold greater than in non-reproductive males (figure 1*e*; two-sample *t*-test, *t*_1,14_ = −12.22, *p* < 0.001). Such increases in sonic muscle size enables acoustic communication and sustained vocal activity during the breeding season, which facilitates a range of social behaviours, including reproduction (e.g. midshipman courtship communication; figure 1*b*). To determine if seasonal hypertrophy of the sonic muscles results in seasonal differences in swim bladder morphology, non-reproductive and reproductive males were microCT scanned. Morphometric analysis following microCT scanning revealed that sonic muscle hypertrophy results in reproductive males having an increased swim bladder width compared with non-reproductive males (figure 1*f*; two-sample *t*-test, *t*_1,14_ = −2.36, *p* = 0.034). Additionally, the enhanced sonic muscles of reproductive males result in a decreased rostral horn length (figure 1*g*; two-sample *t*-test, *t*_1,14_ = 6.71, *p* < 0.001), which subsequently leads to a greater swim bladder to saccular otolith (i.e. sagitta) distance (figure 1*h*; two-sample *t*-test, *t*_1,14_ = −10.28, *p* < 0.001; non-reproductive: 3.31 ± 0.75 mm; reproductive: 11.62 ± 1.57 mm; mean ± s.d.). To assess more subtle morphological variabilities than could be directly measured, the outer surface of non-reproductive and reproductive male swim bladders were pseudo-landmarked (*n* = 2483; electronic supplementary material, figure S3*c*) and compared via PCA. PCA revealed significant differences in non-reproductive and reproductive male swim bladders along PC1, which accounted for 53.5% of overall swim bladder variation (electronic supplementary material, table S1) and corresponded to the greatest variation in the morphology of the rostral horns and the caudal most region of the swim bladder (figure 1*i*).

Similar to previous studies that have investigated reproductive state-dependent differences in male saccular hair cell auditory sensitivity [35,52], we observed that the magnitude of reproductive male saccular auditory evoked potentials in response to pure tone stimuli (95–950 Hz) was greater than in non-reproductive males across a range of biologically relevant iso-intensities (figure 2*d*; one-way repeated measures ANOVA, 154 dB re: 1 µPa: *F*_1,460_ = 30.9, *p* < 0.001; 142 dB re: 1 µPa: *F*_1,450_ = 4.0, *p* = 0.046; 130 dB re: 1 µPa: *F*_1,450_ = 4.7, *p* = 0.031), with frequency-specific differences observed up to 475 Hz. This dramatic increase in auditory evoked potential magnitude resulted in the auditory saccular thresholds of reproductive males being lower (i.e. more sensitive) than non-reproductive males (figure 2*e*; one-way repeated measures ANOVA, *F*_1,404_ = 25.7, *p* < 0.001), with frequency-specific differences in auditory saccular sensitivity observed at 95, 190, 285, 380 and 570 Hz (*a priori t*-tests for pairwise comparison males of different reproductive states across frequency, *p* < 0.05) that resulted in an approximate twofold difference in tuning (figure 2*f*). Furthermore, reproductive state-dependent differences in the highest detectable frequency was observed, with reproductive males exhibiting greater frequency bandwidth sensitivity than non-reproductive males at frequencies ranging from 665–950 Hz (figure 2*g*).

Given the observed seasonal differences (figure 2) and that multiple mechanisms have been shown to result in reproductive state-dependent differences in midshipman auditory sensitivity [32–35,52,53], we tested for seasonal effects of swim bladder morphology on sound pressure sensitivity by comparing the evoked potential magnitudes (*μ*V) and auditory sensitivity (dB re: 1 µPa) of hair cells within the saccule of males of the same reproductive state with intact, sham ablated (i.e. control) or ablated (i.e. removal) swim bladders. When comparing the auditory-evoked potentials from saccular hair cells of non-reproductive and reproductive males with intact or removed swim bladders, it was observed that evoked potential magnitudes were significantly lower among swim bladder removal individuals. However, these differences were greatest among non-reproductive males, which showed significant decreases in the magnitude of their auditory evoked responses across a range of biologically relevant iso-intensities (electronic supplementary material, figure S5*a*; one-way repeated measures ANOVA, 154 dB re: 1 µPa: *F*_1,460_ = 62.2, *p* < 0.001; 142 dB re: 1 µPa: *F*_1,450_ = 24.5, *p* < 0.001; 130 dB re: 1 µPa: *F*_1,450_ = 13.1, *p* < 0.001), with frequency-specific differences that resulted in up to a 12, 11 and sixfold difference in evoked potential magnitude at 154, 142 and 130 dB re: 1 µPa, respectively. By contrast, only subtle differences were observed among reproductive males at the highest intensity tested (electronic supplementary material, figure S5*b*; one-way repeated measures ANOVA, 154 dB re: 1 µPa: *F*_1,460_ = 13.2, *p* < 0.001; 142 dB re: 1 µPa: *F*_1,460_ = 2.9, *p* = 0.091; 130 dB re: 1 µPa: *F*_1,460_ = 4.2, *p* = 0.52).

**Figure 3.**
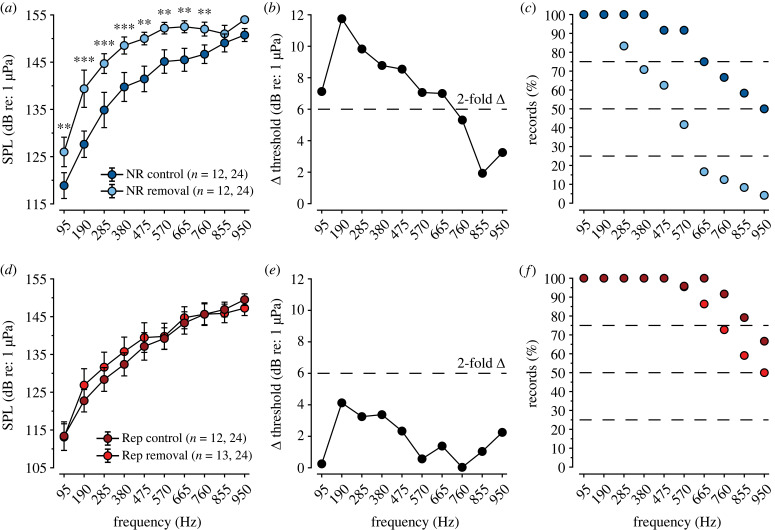
The swim bladder affords greater saccular auditory sensitivity and enhanced frequency bandwidth in non-reproductive males but not reproductive males. (*a,d*) Sound pressure level (dB re: 1 µPa) auditory threshold tuning curves recorded from non-reproductive (*a*) and reproductive (*d*) male midshipman saccular hair cells with intact (dark colours) or ablated (light colours) swim bladders. All data are plotted as mean ± 95% confidence interval. Asterisks indicate significant differences across frequencies: *p < 0.05, **p < 0.01, ***p < 0.001. The number of animals and recordings for each group are indicated in parentheses. (*b,e*) Saccular hair cell threshold difference (Δ dB re: 1 µPa) between male midshipman with intact and ablated swim bladders. The dashed line indicates a twofold change in auditory sensitivity. (*c,f*) Percentage of evoked potential recordings that displayed thresholds above background noise levels across the bandwidth of test frequencies for male midshipman with intact (dark colours) and ablated (light colours) swim bladders.

Similarly, differences in auditory saccular sensitivity relative to sound pressure were only observed among non-reproductive males, with control males exhibiting lower (i.e. more sensitive) auditory thresholds than males that have had their swim bladder removed (figure 3*a*; one-way repeated measures ANOVA, *F*_1,300_ = 29.4, *p* < 0.001) with frequency-specific differences observed between 95–760 Hz (figure 3*a*; *a priori* *t*-tests for pairwise comparisons of non-reproductive control and removal males across frequency, *p* < 0.05) that resulted in a 7–12 dB re: 1 µPa difference in auditory saccular sensitivity (figure 3*b*). Additionally, the removal of non-reproductive male swim bladders resulted in a decreased bandwidth of detectable frequencies, with swim bladder removal males displaying lower detection rates at frequencies ranging from 285–950 Hz (figure 3*c*). However, in contrast, there were no differences in the auditory saccular sensitivity (figure 3*d* and figure 3*e*; one-way repeated measures ANOVA, *F*_1,412_ = 0.6, *p* = 0.42) or bandwidth of detectable frequencies (figure 3*f*) among reproductive control and removal males.

**Figure 4.**
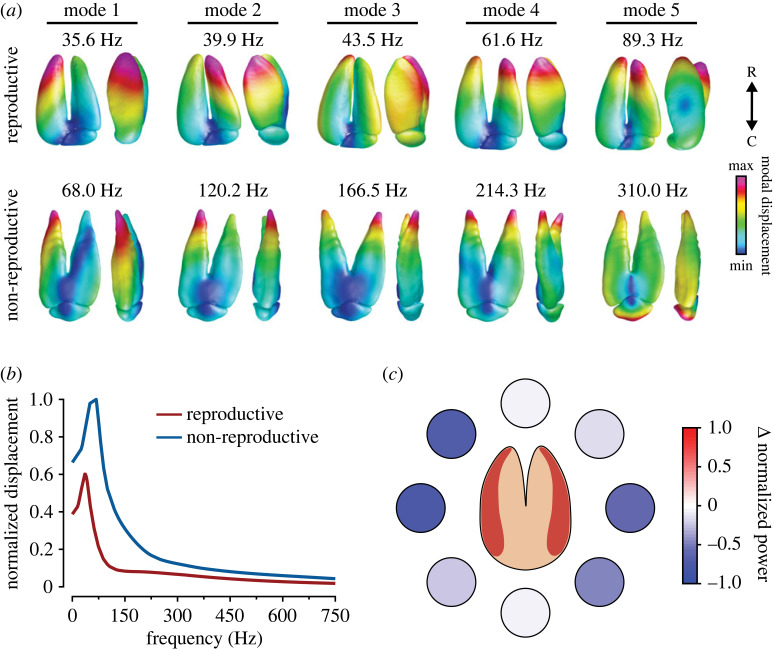
Model of swim bladder response to pressure inputs. (*a*) Dorsal and lateral views of first five displacement modes for a representative reproductive and non-reproductive swim bladder. The natural frequencies of each mode are displayed above. Note that modal displacement is relative to all points within a given mode for each reproductive state. (*b*) Frequency response function. (*c*) Difference in non-reproductive and reproductive swim bladder power at pressure incident angles from 0° to 315° at 45° increments.

Based on morphometric and electrophysiology results, we hypothesized that the observed seasonal differences in sound pressure sensitivity could be due to differences in the distance between the swim bladder and inner ear or a reduction in swim bladder motion in response to a sound field's pressure wave. Therefore, to investigate these hypotheses, we carried out finite-element analyses on representative non-reproductive and reproductive male midshipman swim bladders (figure 4). Natural frequency analysis of the finite-element models showed that swim bladders of both non-reproductive and reproductive males exhibited the greatest modal displacement around the rostral horns of the swim bladder (figure 4*a*). However, the reproductive male swim bladder had a greater number of natural frequencies below 100 Hz (electronic supplementary material, table S2), which is within the range of the fundamental vocalization frequencies observed during social interactions when compared with the non-reproductive swim bladder [54,55]. In addition, harmonic analysis of these models in response to an impinging pressure between 0° and 315° showed that the relative displacement of the non-reproductive swim bladder was greater than the reproductive swim bladder (figure 4*b*). Furthermore, power analysis of the dynamic response showed that the reproductive swim bladder was less than that of the non-reproductive swim bladder across all incident angles where pressure was applied, indicating that the reproductive swim bladder may transduce less auditory information to the inner ear than the non-reproductive swim bladder (figure 4*c*).

## 4. Discussion

In this study, we investigated if sound pressure sensitivity in type I male midshipman is modulated by seasonal changes in swim bladder morphology and whether such morphometric modifications result in seasonal functional changes of the swim bladder. We show using morphological, electrophysiological and modelling approaches that the swim bladder of type I male midshipman exhibits seasonal, reproductive state-dependent changes in morphology and function for sound production and reception. We demonstrate that non-reproductive males exhibit rostral swim bladder extensions that are absent in reproductive males due to hypertrophy of the sonic muscles. This change in swim bladder morphology among non-reproductive males effectively enhances low-frequency (less than 800 Hz) sound pressure sensitivity by decreasing the swim bladder-inner ear distance. By contrast, reproductive males exhibit enlarged sonic muscles around the swim bladder, which enable the production of advertisement calls but also effectively increases the distance between the swim bladder and inner ear, thereby reducing sound pressure sensitivity. In sum, we show that the type I male swim bladder function is seasonally plastic, thus allowing it to serve as an acoustic organ that mediates both sound production and reception.

The swim bladder of teleost fishes serves the primary function of regulating buoyancy and, in some fishes, also acts as an oxygen reservoir (for review, see [16–18]). Additionally, for a subset of fishes, the swim bladder is adapted to facilitate sound production and/or reception [9–11]. The use of the swim bladder for sound production has been widely described in a number of teleost species, including the plainfin midshipman [18,28,56–58]. Furthermore, the swim bladder may also serve as an accessory hearing organ that facilitates the reception of acoustic stimuli via indirect stimulation of the inner ear by sound pressure [59,60]. In this situation, sound pressure-induced vibrations of the swim bladder, which are produced by impinging sound pressure waves, are reradiated in the form of local particle motion, which can then stimulate the particle motion sensitive inner ear end organs [17,39,40]. The degree of sound pressure sensitivity in fishes with swim bladders is directly related to the proximity, or distance, of the swim bladder and auditory inner ear organs (i.e. saccule, utricle and lagena). Given the diversity of swim bladder-inner ear distance relationships among fishes, it is posited that sound pressure sensitivity falls along a continuum that ranges from fish with highly specialized otophysic connections that connect the anterior wall of the swim bladder to the inner ear via Weberian ossicles (e.g. goldfish, catfish, piranhas and their relatives), to fish with their swim bladder in close proximity but not connected to the inner ear (e.g. squirrel fish and cichlids), to fish with their swim bladder distinctly further from the inner ear (e.g. salmonids), to fish with no swim bladder (e.g. sharks and flatfish) [9]. For fish that have swim bladders in close proximity to the inner ear, enhanced sensitivity to sound pressure (dB re: 1 µPa) and higher frequencies have been observed. However, seasonal-dependent changes in swim bladder morphology and its impact on sound pressure sensitivity have not been considered in prior research.

In our study, we show using microCT scanning that type I male midshipman exhibit seasonal changes in swim bladder morphology. We have shown that non-reproductive males display rostral swim bladder extensions that bring the swim bladder within close proximity (3.31 ± 0.75 mm; mean ± s.d.) to the inner ear saccule, and that reproductive males lack such swim bladder extensions due to hypertrophy of the sonic muscles, which enables courting males to produce long-duration advertisement calls and attract conspecific mates [29,36,54]. Additionally, we show that the seasonal enlargement of the sonic muscles results in approximately a 3.5-fold increase in the swim bladder-inner ear saccule distance (11.62 ± 1.57 mm; mean ± s.d.) when compared with non-reproductive males. We hypothesized that this increase in swim bladder-inner ear distance would reduce saccular sound pressure sensitivity in reproductive males due to the absence of rostral swim bladder extensions and the resultant change in the swim bladder's proximity to the inner ear. Previous studies in female midshipman, which have rostral swim bladder extensions [19,39], and in other teleost species with swim bladders in close proximity (approximately 3 mm) to the inner ear [15,61–63] have shown that auditory sensitivity is enhanced to sound pressure signals and acoustic stimuli above 400 Hz when there is a close coupling between the swim bladder and inner ear (i.e. decreased distance). Thus, the morphology of the male swim bladder coupled with changes in swim bladder proximity to the inner ear in non-reproductive males suggests that such males could potentially be sensitive to sound pressure stimuli.

We subsequently tested the hypothesis that pressure sensitivity is specific to non-reproductive males using a well-established electrophysiological approach [41,42,64,65]. Recordings of saccular hair cell evoked potentials indicated that reproductive males have an auditory sensitivity that is approximately 2.5 times greater than that of non-reproductive males at frequencies < 600 Hz (figure 2*e*). In addition, reproductive males exhibited greater saccular auditory evoked potential magnitudes than non-reproductive males in response to pure tone stimuli across a range of biologically relevant iso-intensity stimuli. These seasonal changes in saccular auditory sensitivity and evoked response properties are likely due, in part, to seasonal fluctuations in steroid hormone levels, which are known to modulate the auditory saccular sensitivity in male and female midshipman [34,42,52,66]. The observed reproductive state and likely related hormone-dependent increase in male auditory sensitivity correlate with the harmonic components contained within type I male midshipman vocalizations used during social behaviours. This enhanced sensitivity likely aids in the detection of aggressive calls and/or neighbouring courting males during the reproductive season [28].

In order to resolve the contribution that the swim bladder affords in terms of sound pressure sensitivity in lieu of seasonal hormone effects, we performed swim bladder removals in both reproductive and non-reproductive males, along with the appropriate controls. We showed that non-reproductive males with intact swim bladders exhibited saccular evoked potential magnitudes that were up to 12.5× greater than that observed in non-reproductive males with swim bladders removed, which indicates an increase in the responsiveness of saccular hair cells to sound pressure signals among type I males with intact swim bladders. Subsequently, we showed that non-reproductive males with intact swim bladders, when compared with non-reproductive males with swim bladders removed, exhibited enhanced auditory sensitivity (∼ 7–12 dB re: 1 µPa) across frequencies from 95–665 Hz (figure 3*a,b*), which also indicates that the observed auditory gain is due to the reception of sound pressure in males with intact swim bladders. By contrast, reproductive males with either intact or removed swim bladders showed no difference in saccular evoked potential magnitudes and saccular auditory sensitivity across the bandwidth of tested frequencies (figure 3*d*), which indicates that reproductive males are not, or minimally, sensitive to sound pressure signals. Given these results, we propose that enhanced auditory sensitivity to sound pressure signals in non-reproductive males may be adaptive for enhanced detection and extraction (i.e. auditory scene analysis) of biologically relevant, low-frequency information from natural ambient sounds in the deep offshore waters they inhabit during the non-reproductive winter. Whereas, the reduction or absence of sound pressure sensitivity in reproductive males may be adaptive, in part, to protect the inner ear from self-induced overstimulation by the vocalizations produced during long-duration (up to 2 h) [29,54], high-intensity (source level up to 155 dB re: 1 µPa) courtship calling periods, similar to what has been suggested in the oyster toadfish [67]. Thus, the morphological change in reproductive male swim bladder morphology may allow for a sustained auditory sensitivity to the complex acoustic environment that males inhabit during the reproductive summer.

Finally, our finite-element analysis of type I male swim bladders showed that non-reproductive male swim bladders exhibit greater relative displacement and power when compared with reproductive male swim bladders. Our analysis revealed that the changes in swim bladder morphology can lead to differential swim bladder activation by impinging sound pressure waves, whereby non-reproductive males exhibit greater displacement in response to a broad range of frequencies (up to 750 Hz) when compared with reproductive males. The model also demonstrated that damping is enough to appreciably damp frequency contribution beyond approximately 100 Hz, indicating most motion is dominated by the lower natural frequencies of the swim bladder. Additionally, we showed that the natural frequencies of the reproductive male swim bladder are shifted toward frequencies close to the fundamental frequencies of male social acoustic signals (i.e. hum, grunt and growl). This shift towards lower frequencies may be adaptive for long-duration calling as the swim bladder is easily excited by the sonic muscles at the dominant frequencies contained within the conspecific social signals, thus making it less energetically expensive to vocalize. By contrast, non-reproductive male swim bladders exhibited natural frequencies that resulted in maximal excitation at frequencies that may be associated with natural ambient sounds that propagate efficiently in deep ocean water environments. To validate our model's findings, future experiments that investigate the response of the inner ear otolithic end organs to sound pressure stimuli, such as that produced and controlled in a standing wave tube-like system, coupled with relatively high speed (approx. 100 fps) X-ray phase contrast imaging to track otolith motion over time [68,69], should be conducted to determine how the inner ear end organs respond to both sound pressure and particle motion signals.

In sum, we show that the swim bladder of type I male midshipman is functionally suited to seasonally enhance sound production and mate attraction in reproductive males and serve as an accessory hearing organ to enhance sound pressure sensitivity of the saccule in non-reproductive males. While the seasonal-dependent changes in swim bladder morphology and function observed here have not previously been considered, it is likely that similar adaptions may also occur in other fishes.

## Data Availability

All data and custom MATLAB and R code used in this article are available on Dryad at https://doi.org/10.5061/dryad.ffbg79d1t [70] and GitHub at https://github.com/LoranzieRogers/SwimBladderFunctionalPlasticity. Supplementary material is available online [71].
